# Analysis of Magneto-Optical Hysteresis Loops of Amorphous and Surface-Crystalline Fe-Based Ribbons

**DOI:** 10.3390/ma14010141

**Published:** 2020-12-31

**Authors:** Ondřej Životský, Dmitry Markov, Kamila Hrabovská, Jiří Buršík, Yvonna Jirásková

**Affiliations:** 1 Department of Physics, Faculty of Electrical Engineering and Computer Science, VŠB—Technical University of Ostrava, 17. Listopadu 2172/15, 708 00 Ostrava-Poruba, Czech Republic; markovdmitry94@gmail.com (D.M.); kamila.hrabovska@vsb.cz (K.H.); 2 CEITEC IPM, Institute of Physics of Materials, AS CR, Zizkova 22, 616 00 Brno, Czech Republic; bursik@ipm.cz (J.B.); jirasko@ipm.cz (Y.J.)

**Keywords:** planar flow casting, ribbons, surface magnetism, magneto-optical Kerr microscopy, magnetic force microscopy, magnetic domains, hysteresis loops

## Abstract

Three Fe-based ribbon-type samples prepared by a conventional planar flow casting process are studied from the viewpoint of the amorphous Fe_80_Si_4_B_16_ and partially surface crystallized Fe_80_Si_10_B_10_, and Fe_80.5_Nb_6.9_B_12.6_, microstructures. Surface magnetic properties are investigated by magneto-optical Kerr microscopy, allowing the measurement of a local hysteresis loop from a selected area on the ribbon surface, and simultaneously, a domain structure corresponding to a definite point at the loop. For an amorphous sample, the changes in the slopes of hysteresis loops are related either to the size of the selected surface area, from which the loop is measured, or to the type, width, and movement of magnetic domains through this area. In the first case, the resizing of the area simulates an effect of changing the diameter of the incident laser beam on the magneto-optical properties of the ribbon. In the latter case, the observed wide-curved and fingerprint domains are responsible for markedly different shapes of the hysteresis loops at lower magnetic fields. If the surface is crystallized, the magnetic properties are more homogenous, showing typical one-jump magnetization reversal with less dependence on the size of the surface area. The magneto-optical experiments are completed by transmission electron microscopy and magnetic force microscopy.

## 1. Introduction

There is still increasing interest in the surface magnetic behavior of the materials in light of both fundamental research and applications [1]. Research around the world has concentrated on structurally different materials that are comprehensive and amorphous, ranging from the nanocrystalline up to microcrystalline. Predominantly, the microstructure and the physical properties of amorphous and nanocrystalline materials are of foremost interest from a viewpoint of their surfaces. Among them belong the ribbons prepared by the planar flow casting (PFC) technique [2], whether amorphous or nanocrystalline. Their whole thickness ranges in tens of micrometers, including two structurally different surfaces, each in thickness of several tens of nanometers. The surface being in contact with the surrounding atmosphere and denoted as air-side is shiny and visibly smoother, contrary to the rough opposite wheel-side, which is in contact with the cooling wheel influencing its surface profile. The surface properties can play a key role in the bulk properties [3,4,5] and influence the giant magnetoimpedance (GMI) effect at higher frequencies [6], which are important for the construction of sensors based on the GMI [7,8] and the magnetoelastic [9,10]. Therefore, the surface microstructure in close connection with physical properties are objects of frequent studies using various surface-sensitive methods. Next to the scanning and transmission electron microscopies, or conversion electron Mössbauer spectrometry, the frequently-used non-destructive techniques for the surface magnetic characterization is the magneto-optical Kerr effect (MOKE). It is based on the change of the polarization of incident polarized light when reflected from the surface of the magnetized material. The light penetration depth in metals is only about several tens of nanometers. It is a reason why this technique is highly effective also in investigations of thin-film multilayer systems [11]. Another important feature is the ability to distinguish the contributions from different depths or from different materials present in the system under investigation [12,13]. Due to the phase difference and additivity of magneto-optical effects, all detected contributions can be visualized on the measured hysteresis loop and separated using the linear matrix algebra.

Magneto-optical techniques were successfully applied in the surface studies of the CoFeSiB [14], FeSiB [15], or Fe-Nb-B [16] ribbon-type materials; the magnetically hard nanoparticles embedded in the amorphous matrix were detected on the wheel-side of the Fe_80.5_Nb_6.9_B_12.6_ ribbon [13]. The consequence of partial surface crystallization was the formation of a bias field that shifted the hysteresis loops of strained Fe-Nb-B ribbons [17]. In the present study, the magneto-optical method is concentrated on the selected surfaces of ribbon-type samples with the aim to explain different contributions detectable at MOKE loops and to compare the results from the viewpoint of different microstructures, amorphous and crystallized. 

The surface-amorphous Fe_80_Si_4_B_16_ and surface-crystalline Fe_80_Si_10_B_10_ and Fe_80.5_Nb_6.9_B_12.6_ ribbons were used for the present magneto-optical studies and were partially supported by magnetic force microscopy (MFM). Recently, magneto-optical Kerr microscopy has been applied in such a settlement that the illuminated area on the ribbon surface is shown, and simultaneously, the MOKE hysteresis loop scans over the selected area and reflects the observed magnetic domain structure. The hitherto experiences indicate that the shape of the loop during the magnetization reversal strongly depends on the size of area from which the loop is measured and on the type of domain structure located in this area. 

## 2. Experimental Details

The Fe_80_Si_4_B_16_, Fe_80_Si_10_B_10_, and Fe_80.5_Nb_6.9_B_12.6_ samples were prepared using planar flow casting (PFC) procedures resulting in ribbons of structurally different surfaces. The ribbon surfaces are denoted as AS (air-side) and WS (wheel-side). The WS surfaces were found to be too rough for both surface-sensitive experiments, magneto-optical Kerr microscopy (MOKM), and magnetic force microscopy (MFM), and therefore, only the AS surfaces at all samples were examined. The Si-containing samples were 10 mm wide and 20 µm thick, while the sample containing Nb was 6.5 mm wide and 28 µm thick. 

The amorphous structure and possible traces of crystallization were checked by X-ray diffraction (XRD) using an X’Pert Pro diffractometer (Malvern, Panalytical, Great Britain) with Co-Kα radiation (λ = 0.17902 nm) at room temperature (RT). For the same reason, conversion electron Mössbauer spectrometry (CEMS), with a penetration depth of about 200 nm, was used as well. Measurements were carried out by home-made equipment (Czech Republic) at room temperature (RT) using a ^57^Co(Rh) source. The calibration of velocity scale was performed with α-Fe, and the isomer shifts were given with respect to the RT Mössbauer spectrum of α-Fe. The morphology of samples was followed by a LYRA 3XMU FEG/SEM (TESCAN, Brno, Czech Republic) scanning electron microscope (SEM) at an accelerating voltage of 20 kV, equipped with an X-Max80 detector (Oxford Instruments, High Wycombe, Great Britain) for energy-dispersive X-ray (EDX) analysis. The structural details were obtained by a CM12 STEM (Philips, Eindhoven, The Netherlands) transmission electron microscope (TEM) using 120 kV accelerating voltage. 

The bulk magnetic characteristics, coercivity, saturation, and remanent magnetizations, were determined from hysteresis loops measured using a vibrating-sample magnetometer VSM EV9 (MicroSense, Lowell, MA, USA) at RT on 1.7 cm long ribbons and at an applied magnetic field of about 20 kA/m.

**Figure 1:**
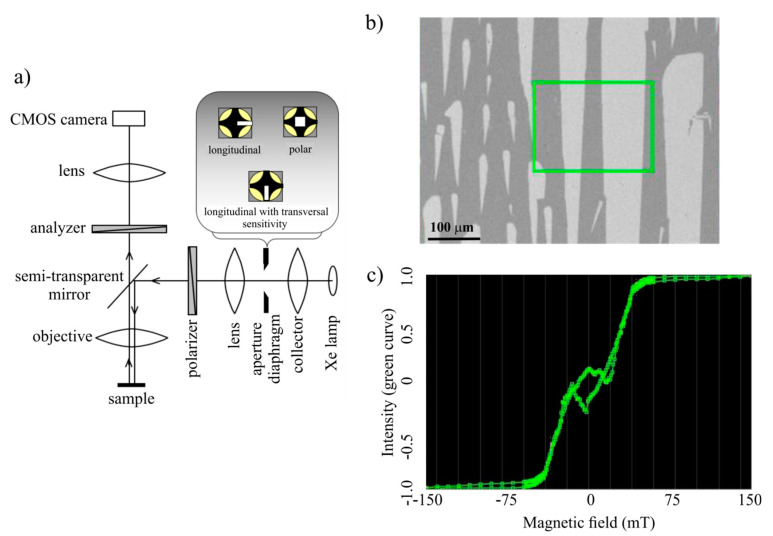
(**a**) Schematic representation of the optical part of a magneto-optical Kerr microscope. (**b**) Observed magnetic domain patterns and choice of sample surface area using the region of interest (ROI) tool (green rectangle). (**c**) Surface hysteresis loop corresponding to the selected surface area.

The surface magnetic properties were studied by MOKM and completed by MFM. MOKM was used for the magnetic domain observations in a static magnetic field and for measurements of the surface hysteresis loops. A schematic representation of Kerr microscope AxioImager M1 (Zeiss, Jena, Germany) is seen in Figure 1a. White light from a Xe lamp passes through an optical system with a polarizer and falls at an oblique angle on a sample placed in a magnetic field *H*. Because magnetization lies mostly in the plane of the ribbon due to its dimensions, the sensitivity was set up to the in-plane longitudinal magnetization component *M_L_* (parallel to the plane of light incidence and applied magnetic field) using the aperture diaphragm in the back focal plane of microscope. The light reflected from the sample goes through the analyzer almost crossed with the polarizer and is finally captured by a CMOS camera. The magnetic domain relief (Figure 1b) is recorded in real time. It is obtained by a subtraction of images; the first one is taken from the ribbon surface at an applied saturation magnetic field, and the next images are taken from the same place at a decreasing magnetic field and are subsequently subtracted from the first one. In such a way, a series of magnetic domain reliefs are obtained. To get the best contrast of the final domain pattern, all results are averaged. A magneto-optical hysteresis loop (Figure 1c) shows the dependence of the averaged Kerr intensity, normalized to the maximum of the absolute intensity value resulting in ±1 range, on an applied magnetic field. It is taken from the illuminated selected area and defined using the region of interest (ROI) tool (the green rectangle in Figure 1b). The first point of the loop is measured in a negative saturation magnetic field, and a corresponding magnetic domain figure is stored. Afterwards, the magnetic field is reduced by a predefined step and the domains are obtained again. In this way, positive saturation is achieved firstly, followed by a return to negative saturation. Finally, the output of the used KerrLab software shows the surface hysteresis loop and magnetic domain patterns measured at each point of the loop. The resolution of domain patterns is slightly lower compared to MFM in consequence of the optical microscope used. 

MFM experiments were done at RT under an ambient atmosphere by Ntegra Prima microscope (NT-MDT, Moscow, Russian Federation) using a commercially available MFM10 probe with CoCr 40 nm coating in a semi-contact mode. The magnetic domain visualization was performed by a two-pass method. The surface topography was obtained by a tapping mode in the first step and the magnetic contrast by a lifting of the probe above the surface in the second step without an applied external magnetic field. The maximum size of the analyzed area was 100 × 100 µm^2^. 

## 3. Results and Discussion

### 3.1. Microstructural and Bulk Magnetic Properties

**Figure 2:**
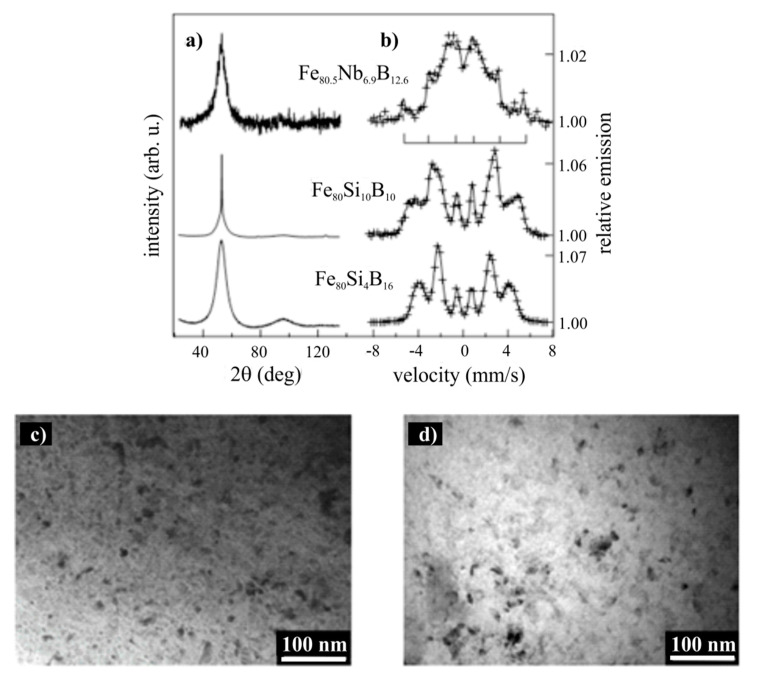
XRD patterns (**a**) and conversion electron Mössbauer spectra (**b**) taken from the air-side of Fe_80_Si_4_B_16_, Fe_80_Si_10_B_10_, and Fe_80.5_Nb_6.9_B_12.6_ samples. TEM images of the air-side microstructure for Fe_80_Si_10_B_10_ (**c**) and Fe_80.5_Nb_6.9_B_12.6_ (**d**) samples.

The X-ray diffraction patterns taken from the air-sides of all samples in the as-quenched states are shown in Figure 2a and corresponding CEMS spectra in Figure 2b. The amorphous structure is observed only at the Fe_80_Si_4_B_16_ sample. This was reflected in two broad halos in a diffractogram and in a broad six-line spectrum (+) analyzed by a superposition of two distributions of hyperfine inductions (not presented here), resulting in the full line spectrum. The other two samples, Fe_80_Si_10_B_10_ and Fe_80.5_Nb_6.9_B_12.6_, were partially crystallized. It was documented by small sharp peaks superimposed on a broad amorphous halo in diffractograms and additional component(s) in the Mössbauer spectra. The components represented by sextets with sharp lines, better seen in the spectrum of the Fe_80.5_Nb_6.9_B_12.6_ sample, were attributed to the crystalline grains which are embedded in the amorphous matrix in the close surface layers corresponding to depth sensitivity of CEMS measurement. One of the six-line components with hyperfine induction around 33 T was depicted as a line plot between both crystalized samples. The small crystallites are clearly visible in the TEM images (Figure 2c,d). The intergranular amorphous matrix was subjected to substantial microstructural and chemical changes due to its enrichment in elements which were expelled from the crystalline precipitates [18]. 

It is also worth noting that the initial amorphous structure was not a simple system in terms of the local topological and chemical ordering, metastability, and heterogeneity. This directly influenced the magnetic properties, and the short-ordered species acted as centers of crystallization. These inhomogeneities were reflected, e.g., in the asymmetry of the Fe_80_Si_4_B_16_ CEMS spectrum (Figure 2b) and were detected by other experimental observations. Predominantly, High-Resolution Transmission Electron Microscopy (HRTEM) [19] enabled the observation of local structural organizations on an atomic scale, consisting of small localized short-ordered regions a few nanometers in size. The mean size of these species or clusters can be calculated from the position of the, e.g., first XRD broad peak and its full width at half maximum (FWHM). The mean size obtained from the presented diffractogram of the Fe_80_Si_4_B_16_ sample yields the value 1.4 nm, well corresponding with theoretical calculations of atomic and cluster ordering in amorphous Fe-B alloys [20].

**Figure 3:**
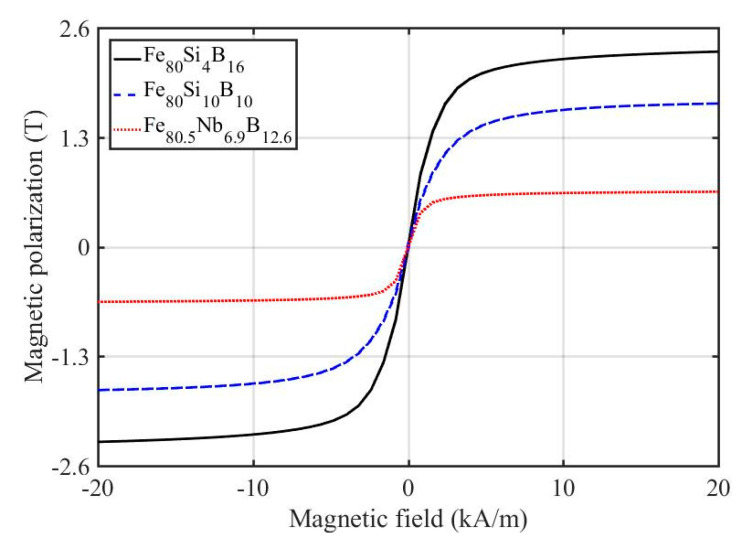
Bulk hysteresis loops of analyzed ribbons measured using the vibrating-sample magnetometer.

Bulk hysteresis loops of all studied samples measured using the VSM are shown in Figure 3, and the analyzed magnetic parameters, i.e., saturation, *J_s_*; remanent, *J_r_*; magnetic polarizations; and coercivity, *H_c_*, are shown in Table 1. The best soft magnetic properties, the highest saturation magnetic polarization (2.32 T), and the lowest coercivity (≈15 A/m), were detected for the amorphous Fe_80_Si_4_B_16_ ribbon. The crystallization of the other two samples was manifested by an increase in coercivity to 21 A/m (Fe_80.5_Nb_6.9_B_12.6_) and to 50 A/m (Fe_80_S_i10_B_10_). 

### 3.2. Surface Magnetic Properties—Amorphous Fe_80_Si_4_B_16_ Ribbon 

**Figure 4:**
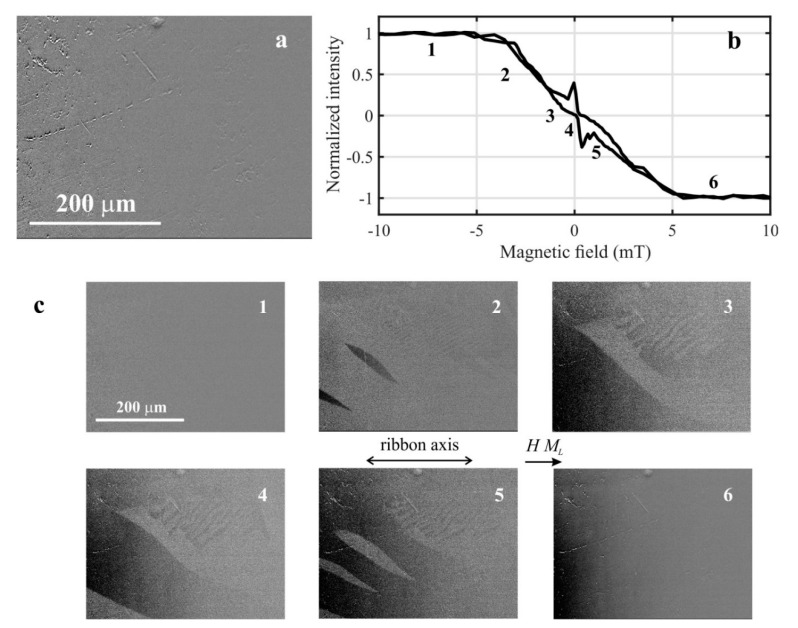
Selected illuminated area on the air-surface of amorphous Fe_80_Si_4_B_16_ ribbon (**a**), normalized magneto-optical hysteresis loop (**b**), and consecutive magnetic domain patterns (1 to 6) obtained during switching magnetic field from negative to positive saturation (**c**).

**Figure 5:**
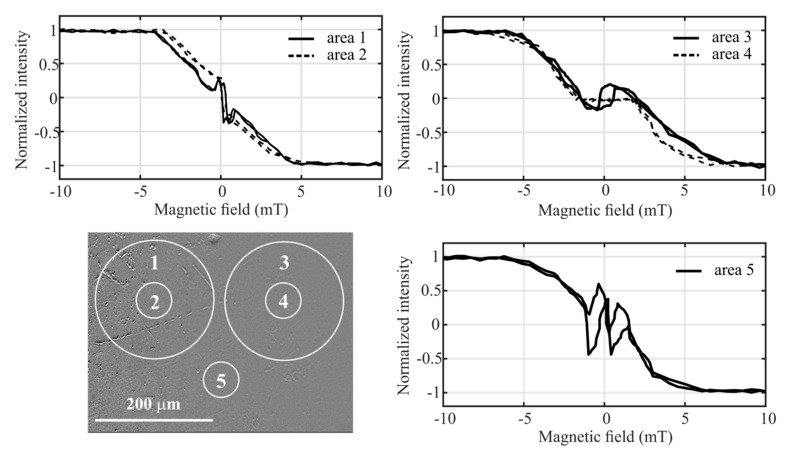
Magneto-optical hysteresis loops of amorphous Fe_80_Si_4_B_16_ ribbon obtained by averaging over different surface areas denoted by white circles with diameters 200 μm (areas 1 and 3) and 60 μm (areas 2, 4, and 5).

Magnetic domains and magneto-optical hysteresis loops taken from the local surface places of the air-side of amorphous Fe_80_Si_4_B_16_ ribbon are shown in Figure 4 and Figure 5, respectively. Figure 4a,c depicts the selected illuminated surface area of dimensions 450 × 340 μm^2^ (using an objective with magnification 20) and magnetic domain patterns during switching the applied magnetic field from −10 mT (negative saturation—light-colored) to +10 mT (positive saturation—dark-colored). The observed wide-curved and fingerprint-like magnetic domains are typical for the as-quenched ribbons, indicating the presence of tensile and planar compressive stresses in corresponding local places. These stresses originate in a production process and/or are due to a subsequent manipulation. They are responsible for the strongly inhomogeneous surface magnetic properties. As a result, the shape of the measured magneto-optical hysteresis loop obtained by averaging the Kerr intensity over the whole illuminated area is untypical (Figure 4b), and the magnetization reversal reflects the presence of both types of domains on the ribbon surface. The domain structure inhomogeneity causes change in the shape of the magneto-optical hysteresis loop by illumination of another place on the ribbon surface.

Figure 5 shows the same illuminated area as in the previous case, but the obtained hysteresis loops were averaged over smaller white circular areas with diameters of 200 μm (areas 1 and 3) and 60 μm (areas 2, 4, and 5). These areas were selected by means of a region of interest (ROI) tool. Resizing of the averaged area simulates an effect at which a change of the incident laser beam diameter influences the magneto-optical properties of the sample surface. The results clearly indicate that if the averaged area is reduced, the hysteresis loop in the range of low magnetic fields of about ±4 mT can markedly change its shape due to local magnetic domains distribution. Hysteresis loops obtained by averaging over circles 1 and 2 reflect gradual origin, movement, and extension of wide band domains rising in this part of the sample. In both cases, the magnetic domains arise at a magnetic field of about −4 mT, and the intensity nearly linearly decreases with the decreasing negative magnetic field. The difference occurs at a magnetic field of −0.5 mT, when area 2 is completely covered by the dark domain(s). This leads to a fast magnetization reversal. By contrast, in the larger area 1, a short increase in intensity is observed. This effect can be explained by short-time narrowing of the band domain(s) passing through area 1 and causing an average increase of light color inside it. This is followed by a typical magnetization reversal.

A different shape of the hysteresis loops is observed in areas restricted by circles 3 and 4 through which the fingerprint domains pass. At low magnetic fields, approximately ±1.5 mT, a nearly constant magneto-optical response without visible reversal is observed inside area 4. It means that in such a small area, the movement of the fine domains is practically not observable. Nevertheless, if the area is enlarged (area 3), the magnetization reversal is measured also in a case of the fine fingerprint domains. The number of the fine light-colored domains here dominates over that of dark-colored ones. This leads to the negative reversal observed on the measured loop. By the subsequent increase of the magnetic field, the domains become denser, and the magnetization continues in its original slow reversal. The hysteresis loop taken from the area restricted by the small circle 5 is characterized by multiple jumps during the magnetization reversal due to the alternating widening and narrowing of the crossing band domains.

The presented examples of hysteresis loops are similar to those measured on the thin multilayer systems (see, for example, [12]). Here, the authors introduced an ability of magneto-optics to distinguish two or more magnetically different phases of dissimilar anisotropies on the measured loop and called it depth and/or material sensitivity [13]. In the case of the amorphous Fe_80_Si_4_B_16_ ribbon, the composed loops could theoretically come from the nanosized FeSi and FeB species (clusters) formed as a consequence of topological and chemical inhomogeneities [15]. This cannot be completely ruled out, but the MOKM does not have sufficient resolution thanks to the optical microscope that was used. On the other hand, the results shown in Figure 5 clearly document that the shape of the magneto-optical loop during reversal depends on the size of the area from which the loop is obtained as the type, width, and movement of domains across this area. In reality, magneto-optics uses its material sensitivity here as well, as both types of domains show different magnetic anisotropies and the loop detects their presence if they are located within a selected surface area. That is a reason why the shape of the hysteresis loop will be unique for each place and for a different area selected on the surface of the amorphous Fe_80_Si_4_B_16_ ribbon. Moreover, even without the visualization technique, the shape of the hysteresis loop roughly reflects the type of magnetic domains present on the ribbon surface.

### 3.3. Surface Magnetic Properties—Surface-Crystalline Fe_80_Si_10_B_10_ and Fe_80.5_Nb_6.9_B_12.6_ Ribbons 

**Figure 6:**
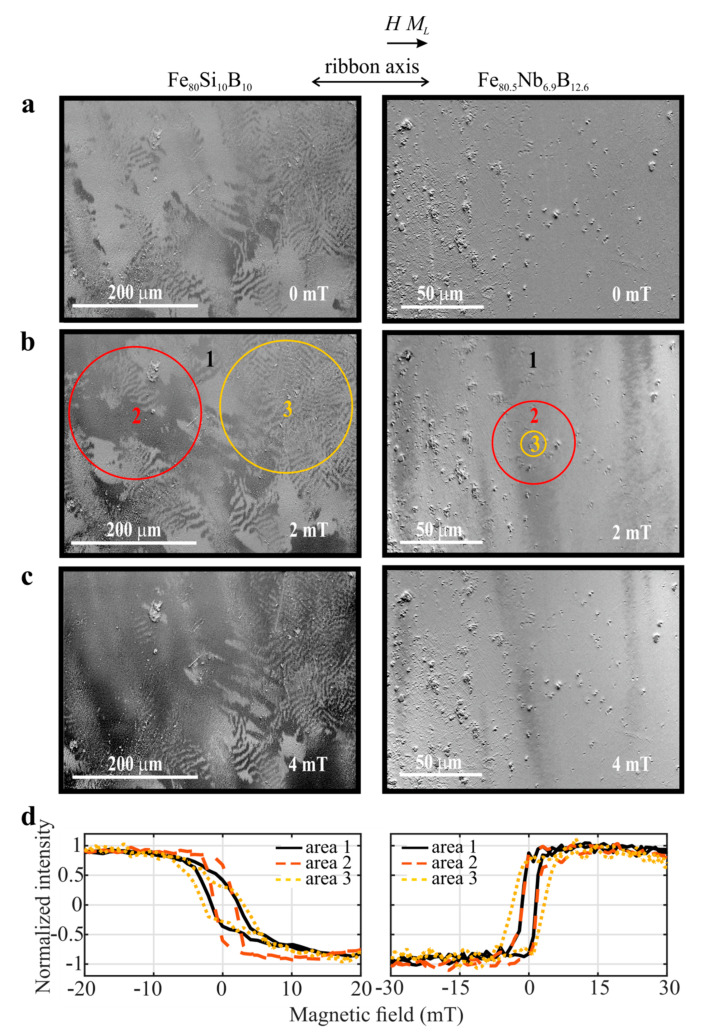
Magneto-optical measurements of the surface-crystalline Fe_80_Si_0_B_10_ (left panel) and Fe_80.5_Nb_6.9_B_12.6_ (right panel) ribbons. Magnetic domain patterns are visualized at applied magnetic fields 0 mT (**a**), 2 mT (**b**), and 4 mT (**c**). Hysteresis loops corresponding to the highlighted surface areas are shown in bottom subplots (**d**).

Results of the magneto-optical Kerr microscopy measurements on the partially crystallized air-surfaces are shown in Figure 6 for the Fe_80_Si_10_B_10_ (left panel) and Fe_80.5_Nb_6.9_B_12.6_ (right panel) samples. The domain structures at both samples are demonstrated in dependence on the magnetic field; a—0 mT, b—2 mT, and c—4 mT. Figure 6d shows the hysteresis loops obtained from the illuminated rectangle area of the size 450 × 340 μm^2^ (curve 1), and from the circle areas of lower diameters highlighted in Figure 6 (curves 2 and 3). The images for the Fe_80_Si_10_B_10_ sample illustrate two kinds of domain patterns; irregular wide-curved domains and narrow fingerprint domains similar to the previous amorphous surface of the Fe_80_Si_4_B_16_ sample. Nevertheless, the behavior of the wide domains during changes of the magnetic field was somewhat different in the surfaces of both samples, whereas in case of the amorphous surface (Section 3.2), the band domains changed their width up to disappearing and newly appearing during a transition of the magnetic field from negative to positive saturation. The band domains of the Fe_80_Si_10_B_10_ sample only enlarge; dark colors increase at the expense of light ones. The difference in the behavior of domains between both surfaces is also reflected in the hysteresis loops. In the first case, the multiple jumps are visible on the hysteresis loop during magnetization reversal. In the second case, a simple reversal of the magnetization is observed. Nevertheless, the shape of the hysteresis loops during the simple reversal also depends on the size and place from which it was taken. The fastest reversal of magnetization and the low coercivity (≈1.5 mT = 1.2 kA/m) was detected at hysteresis loop 2, corresponding to area 2, where mainly band domains are passed through (Figure 6b). By contrast, the slowest magnetization reversal is observed at curve 3, taken from area 3 with dominating fingerprint domains. In this case, the hysteresis loops are clearly composed of two phases. Similar but visibly smaller unevenness is seen on hysteresis curve 1, resulting in higher coercivity (≈1.95 mT), corresponding to the whole rectangle area 1 where both types of domains contribute to its form. 

Results of the magneto-optical measurements done on the crystallized Fe_80.5_Nb_6.9_B_12.6_ ribbon surface are seen in the right panel of Figure 6. They were performed with a 50× magnification objective, which resulted in a reduction of the illuminated area to a rectangle with dimensions 180 × 136 μm^2^ (area 1). In addition, the hysteresis loops were measured also over two smaller circular areas, with diameters of 50 μm and 15 μm (areas 2 and 3), respectively. It can be seen that the magneto-optical contrast and the obtained hysteresis loops are inverted compared to both previous samples. The reason is that a switch-over of the magnetic field from -30 mT to +30 mT corresponds to the transition from dark (negative intensity) to light (positive intensity) color. The surface seems to be much rougher compared to the surface of the Fe_80_Si_10_B_10_ sample. In Figure 6b, the band domains are visible, and at first sight, the fingerprint domains are missing. Additionally, a presence of dominating wide band domains influences the hysteresis loops that are, for areas 1 and 2, practically identical. Nevertheless, the curve taken from the smallest area, area 3, is slightly different, which can reflect finer structure over this area.

**Figure 7:**
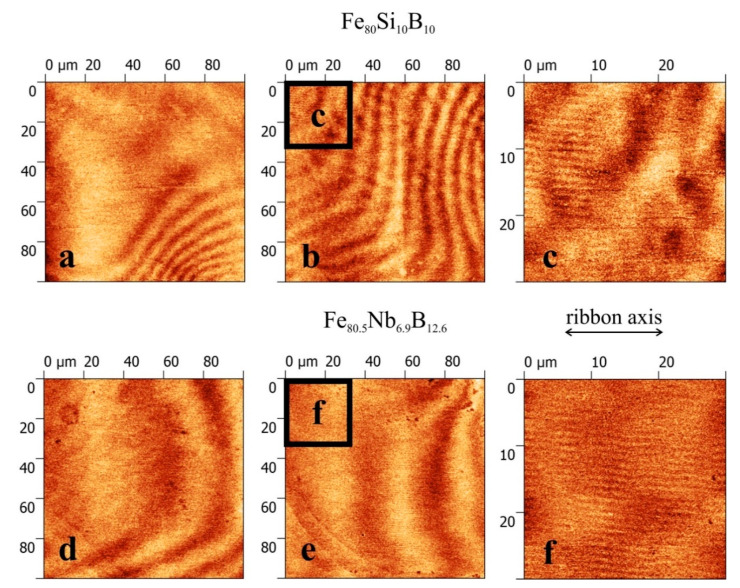
Magnetic force microscopy images of domains observed on the surfaces of Fe_80_Si_0_B_10_ (top) and Fe_80.5_Nb_6.9_B_12.6_ (bottom) ribbons. Patterns are shown at two different surface areas with dimensions 100 × 100 μm^2^ (**a**,**b**,**d**,**e**). Subplots (**c**,**f**) present details (30 × 30 μm^2^) of images (**b**,**e**), respectively.

The illustration of domains for the Fe_80_Si_0_B_10_ (top) and Fe_80.5_Nb_6.9_B_12.6_ (bottom) in Figure 7 was obtained using magnetic force microscopy in a higher resolution. The magnetic tip used at MFM is oriented perpendicular to the sample surface, and thus, it is sensitive to an out-of-plane magnetization component, contrary to the MOKM. Domain patterns from two different surface places of both samples, in Figure 7a,b,d,e, indicate that the domains reflect both the in-plane and out-of-plane magnetization components and that they consist of finer irregular structures clearly visible and in detail in Figure 7c,f corresponding to black squares that are 30 × 30 µm^2^. This cannot be distinguished by the MOKM due to the optical microscope usage. The lines along the ribbon axis visible in the details (Figure 7c,f) are surface artefacts caused by scanning. From the differences in domain patterns between both crystallized surfaces can be deduced a different magnetic behavior of nanocrystallites magnetically coupled by a ferromagnetic metallic glass matrix.

## 4. Conclusions

The present work is devoted to the detailed analysis of the microstructural and magneto-optical properties taken from the air-surfaces of the Fe_80_Si_4_B_16_, Fe_80_Si_10_B_10_, and Fe_80.5_Nb_6.9_B_12.6_ ribbons prepared by a planar flow casting technique. The main conclusions of our study can be summarized in the following points:

(1) XRD diffractograms and CEMS spectra confirmed an amorphous structure at the air-surface of the Fe_80_Si_4_B_16_ sample. The other two samples, Fe_80_Si_10_B_10_ and Fe_80.5_Nb_6.9_B_12.6_, are partially crystallized, as documented by small sharp peaks superimposed on a broad amorphous halo in diffractograms and additional component(s) in the Mössbauer spectra. The small surface crystallites are clearly seen in the TEM images;
(2) The best bulk soft magnetic properties are observed in the case of the anamorphous Fe_80_Si_4_B_16_ ribbon. The crystallization of the other two samples is reflected by an increase in the coercivity;
(3) Magneto-optical properties of the amorphous sample show a strong sensitivity to the type of the surface magnetic domains and to the size of the surface area from which the loop is averaged. In both cases, the measured hysteresis loops exhibit different magnetization reversals at lower magnetic fields. It is experimentally demonstrated that contributions of both wide-curved and fingerprint-like magnetic domains can be distinguished on the measured loop due to the magneto-optical material sensitivity;
(4) The prevailing contribution of the irregular wide-curve domains only enlarging with an increasing magnetic field and causing a simple magnetization reversal without significant dependence on the selected area size was observed in the case of the surface-crystalline ribbons. Magnetic force microscopy evidenced that the domain bands are in reality formed by finer structures. In some places, these domains are combined with the amorphous fingerprint domains, slowing down the magnetization reversal, and both contributions are again visible on the measured magneto-optical hysteresis loop.

## Figures and Tables

**Table 1:** Bulk magnetic parameters of investigated ribbons: *J*_s_—saturation magnetic polarization; *J*_r_—remanent magnetic polarization; *H*_c_—coercive field.

Ribbon	*J*_s_ (T)	*J*_r_ (T)	*H_c_* (A/m)
Fe_80_S_i4_B_16_	2.32	0.02	14.97
Fe_80_S_i10_B_10_	1.70	0.03	49.97
Fe_80.5_Nb_6.9_B_12.6_	0.66	0.01	21.00

## Data Availability

The data presented in this study are available on request from the corresponding author.

## References

[1] Li F.C., Liu T., Zhang J.Y., Shuang S., Wang Q., Wang A.D., Wang J.G., Yang Y. (2019). Amorphous–nanocrystalline alloys: Fabrication, properties, and applications. Mater. Today Adv..

[2] Mattson J., Theisen E., Steen P. (2018). Rapid solidification forming of glassy and crystalline ribbons by planar flow casting. Chem. Eng. Sci..

[3] Cao C.C., Wang Y.G., Zhu L., Meng Y., Zhai X.B., Dai Y.D., Chen J.K., Pan F.M. (2018). Local structure, nucleation sites and crystallization behavior and their effects on magnetic properties of Fe_81_Si_x_B_10_P_8−x_Cu_1_ (x = 0~8). Sci. Rep..

[4] Dong W., Bai G., Yi S., Yan M. (2019). Effect of CO gas on surface profile and magnetic properties of Fe–Si–B amorphous ribbons. J. Mater. Sci. Mater. Electron..

[5] Zhang J., Wan F., Li Y., Zheng J., Wang A., Song J., Tian M., He A., Chang C. (2017). Effect of surface crystallization on magnetic properties of Fe_82_Cu_1_Si_4_B_11.5_Nb_1.5_ nanocrystalline alloy ribbons. J. Magn. Magn. Mater..

[6] Mansourian S., Bakhshayeshi A., Taghavi mendi R. (2020). Giant magneto-impedance variation in amorphous CoFeSiB ribbons as a function of tensile stress and frequency. Phys. Lett. A.

[7] Gazda P., Szewczyk R. (2020). Novel Giant Magnetoimpedance Magnetic Field Sensor. Sensors.

[8] Malátek M., Kraus L. (2010). Off-diagonal GMI sensor with stress-annealed amorphous ribbon. Sens. Actuators A Phys..

[9] Beato-López J.J., Urdániz-Villanueva J.G., Pérez-Landazábal J.I., Gómez-Polo C. (2020). Giant Stress Impedance Magnetoelastic Sensors Employing Soft Magnetic Amorphous Ribbons. Materials.

[10] Hrabovská K., Životský O., Rojíček J., Fusek M., Mareš V., Jirásková Y. (2020). Surface Magnetostriction of FeCoB Amorphous Ribbons Analyzed Using Magneto-Optical Kerr Microscopy. Materials.

[11] Schmidt D., Briley C., Schubert E., Schubert M. (2013). Vector magneto-optical generalized ellipsometry for sculptured thin films. Appl. Phys. Lett..

[12] Postava K., Sveklo I., Tekielak M., Mazalski P., Maziewski A., Stupakiewicz A., Urbaniak M., Szymanski B., Stobiecki E. (2008). Material selective sensitivity of magneto-optical Kerr effect in NiFe/Au/Co/Au periodic multilayers. IEEE Trans. Magn..

[13] Postava K., Hamrle J., Hamrlová J., Hrabovský D., Životský O., Pištora J., Lukáš D. (2012). Depth and material sensitivity in magneto-optic nanostructures. Int. J. Nanotechnol..

[14] Chizhik A., Vega V., Mohamed A., Prida V.M., Sanchez T., Hernando B., Ipatov M., Zhukova V., Zhukov A.P., Stupakiewicz A. (2017). Surface magnetic properties and giant magnetoimpedance effect in Co-based amorphous ribbons. Intermatellics.

[15] Životský O., Hendrych A., Klimša L., Jirásková Y., Buršík J., Gómez J.A.M., Janičkovič D. (2012). Surface microstructure and magnetic behavior in FeSiB amorphous ribbons from magneto-optical Kerr effect. J. Magn. Magn. Mater..

[16] Životský O., Postava K., Kraus L., Jirásková Y., Juraszek J., Teillet J., Barčová K., Švec P., Janičkovič D., Pištora J. (2008). Surface and bulk magnetic properties of as-quenched FeNbB ribbons. J. Magn. Magn. Mater..

[17] Kraus L., Životský O., Postava K., Švec P., Janičkovič D. (2008). Exchange bias in surface-crystalline Fe-Nb-B ribbons. IEEE Trans. Magn..

[18] Inoue A., Takeuchi A., Makino A., Masumoto T. (1996). Soft and hard magnetic properties of nanocrystalline Fe-M-B (M = Zr, Nd) base alloys containing intergranular amorphous phase. Sci. Rep. Ritu A.

[19] Hirotsu A. (1994). High resolution electron microscopy of medium-range order in amorphous alloys. Mat. Sci. Eng. A.

[20] Aykol M., Mekhrabov A.O., Akdeniz M.V. (2009). Nano-scale phase separation in amorphous Fe–B alloys: Atomic and cluster ordering. Acta Mater..

